# Comparative assessment of endogenous lipid transfer protein (LTP) level in genetically modified maize for its relevance in safety assessment

**DOI:** 10.1080/21645698.2026.2615498

**Published:** 2026-01-16

**Authors:** Eric Ma, Saurabh Joshi, Ivan Birukou, Eric Rosenbaum

**Affiliations:** Seeds Product Safety, Syngenta Seeds, Durham, NC, USA

**Keywords:** Allergenicity, genetic modification, GM, lipid transfer protein, LTP, maize, risk assessment

## Abstract

Allergenicity risk has been one of the safety concerns for genetically modified (GM) crops that are generated using modern biotechnologies. When there is the presence of endogenous allergens in a host crop, one question is often raised if genetic modification could increase their allergenicity risk. In this study, lipid transfer protein (LTP), the major endogenous allergen in maize, was measured and compared between various GM maize with their non-GM control as well as non-GM, commercial maize varieties. The results showed LTP levels have no meaningful difference in GM maize, and the LTP levels observed in GM maize were well encompassed within the potential natural variation range from non-GM maize varieties. Therefore, such endogenous LTP quantitation comparison in GM and non-GM control maize does not bring valuable information to the safety assessment of GM maize.

## Introduction

It has been over three decades since the first genetically modified (GM) crop was introduced for human food consumption.^1^^1^U.S. Food & Drug Administration. https://www.fda.gov/food/agricultural-biotechnology/science-and-history-gmos-and-other-food-modification-processes. Over the past decades, many GM crops have been subsequently developed, and prior to acquiring regulatory approval for commercialization, comprehensive and rigorous safety assessments have been conducted to ensure their safe use for human consumption as well as for animal feeds. Currently, the principles and guidelines adopted by Codex Alimentarius Commission have been used as the foundation of the scientific guidelines for the risk assessments of GM food crops over decades around the world.^1,2^ One critical concern in the assessment of GM crop safety is regarding any potential food allergy that the genetic modification may introduce into or alter in the resulting GM crops.^3,4^ From common food staples such as rice, wheat, soybean, maize, barley, etc., there are endogenous proteins that are considered food allergens.^5^ For example, soybean [*Glycine max* (L.) Merr.] is listed as a major allergy food in many countries^6,7^; hence, its endogenous allergen proteins are required to be evaluated when genetic modification is applied to soybean.^6,8^ For maize or corn (*Zea mays* L.), an endogenous protein, lipid transfer protein (LTP) is considered the major maize allergen protein.^9,10^ Unlike the commonly seen soybean allergy, maize has a long history of safety food and feed uses, and it is not considered an allergy food around the world.^11^ However, when genetic modification is applied to maize, questions were raised about whether or not the LTP levels would be elevated, leading to increased allergenicity risk in GM maize.

Maize is genetically modified by transformation to generate transgenic “events” to convey the desired trait, and a well-characterized event (a new variety) with the best combined trait efficacy and safety profile will be advanced to regulatory approval and subsequent commercialization.^12^ These single-event varieties are usually bred to create new varieties with “stacked” events via conventional breeding (e.g., cross-pollination).^13^ In this study, we examined the endogenous LTP levels in both single-event and stacked-event GM maize varieties. The endogenous LTP levels in GM maize varieties also compared with those from non-GM, conventional maize varieties to assess any potential impact of genetic modification on them, and subsequently whether or not endogenous LTP analysis would contribute any value to the safety assessment of GM maize.

## Materials and methods

### Materials

*Maize kernel* was used for the analysis of LTP content. Kernel samples were harvested from GM maize hybrid varieties and their respective non- transgenic, near-isogenic counterpart maize variety that was produced in the same field trials. In addition, commercially available, conventional varieties were also included as references. The plants were grown at locations that were in typical maize cultivation areas following the regional agronomic practice for planting, maintenance, and harvesting.^14^

*Purified LTP and antibodies*: Maize endogenous LTP was purified from conventional maize kernels using cation exchange followed by size-exclusion chromatography. For Western blot analysis, 200 ng of the purified LTP was loaded onto polyacrylamide gel for use as a standard. The presence of the LTP in Western blot was probed with a rabbit anti-LTP antibody (Pacific Immunology. Ramona, CA) and then detected with an alkaline phosphatase-conjugated donkey anti-rabbit secondary antibody.

*Maize kernel extracts for Western blot analysis*: The kernels from GM maize, control, and commercial reference varieties were ground and extracted with an extraction buffer. In brief, the ground kernel powder was suspended in 25 mM sodium acetate buffer (pH 4.0) and then incubated at 5°C ±3°C with shaking overnight. The mixture was centrifuged and the supernatant was filtered to remove solids. The crude extract was first analyzed for its total protein concentration via bicinchoninic acid (BCA) assay using bovine serum albumin as the protein standard prior to loading onto sodium docecyl sulfate-polyacrylamide gel electrophoresis (SDS-PAGE) gels the equal amounts of total proteins from the various maize varieties to be bested.

### Assay methods

*Western Blot analysis*: For a semi-quantitative comparison, the protein extracts from various maize varieties were first analyzed by BCA assay to determine its total protein concentration. Here, 5 μg of total protein from each extract sample was loaded along with 200 ng of purified LTP standard to an SDS-PAGE gel for separation, and subsequently transferred to a Western blot membrane. After antibody binding and washing, an alkaline phosphatase substrate solution was used for the LTP band visualization on the membrane in order to compare their relative levels from the maize samples. Each Western blot was performed in duplication.

#### Mass Spectrometry Analysis

Maize LTP contents were quantified by LC-MS/MS method, which was adapted from a previous published work.^15^ Briefly, a multiple reaction monitoring (MRM) assay was conducted using a triple quadrupole mass spectrometer coupled to ultra performance liquid chromatography (UPLC). In the MRM assay, the protein fraction was extracted from maize kernel samples, followed by reduction/alkylation of disulfide bonds and subsequent digestion by trypsin. The concentrations of surrogate peptides unique to target LTPs (Table 1) were selectively quantitated by comparing their MS signals to those of the internal standard peptides added to sample digests at fixed, known concentrations. The internal standard peptides [stable isotopically labeled peptides (SILs)] are identical in amino acid sequence to the surrogate peptides of the LTP proteins, except for the heavy isotope labeling of the C-terminal lysine, which distinguishes the standards from the surrogate peptides by a heavier mass (Table 1). The ratio of the MS signals of surrogate LTP peptide to the internal standard is equal to the ratio of the corresponding peptide concentrations (e.g., LTP peptide/standard) and, thus, can be converted to the concentration of the surrogate LTP peptide since the concentration of the internal standard is known. Finally, the concentration of each LTP protein is extrapolated from the concentrations of its unique surrogate peptides, and the total LTP concentration is calculated by summing all measured LTP variants.

**Table 1.** LTP proteins and respective surrogate peptides.

Protein abbreviation	Full protein name	Protein description	Molecular weight (g/mol)	Surrogate peptide*
Zea m 14^#^	sp | P19656 (sp56)	phosphoLTP1	12,659.23	NAAAGVSGLNAGNAASIPSK
LTPa^#^	GRMZM2G101958 (GR58)			
LTPb	GRMZM2G010868 (GR68)	LTP	11,718.82	GVSGLNAGNAASIPSK
LTPc	GRMZM5G898755 (GR55)	nonspecificLTP4pre	13,319.46	LGGGVSMANAANIPSK

Table 1 lists the surrogate peptide sequences for quantitation of total maize LTP proteins in mass spectrometry analysis. * The last lysine of each surrogate peptides was radio-labeled with C13N15-heavyK as the internal standard (SIL). ^#^Zea m 14 allergen is a variant of the GRMZM2G101958 gene locus. Therefore, both Zea m 14 and LTPa proteins were quantitated together using a single surrogate peptide.

For the early MRM assay method employed in the maize event MZIR098 study in 2014, samples were prepared following the procedure developed by Stevenson *et al*.^15^ Specifically, maize samples were homogenized and extracted in 1:1 mix of sucrose buffer (0.9 M sucrose, 100 mM Tris-HCl pH 8.0, 10 mM EDTA, 0.4% *β*-mercaptoethanol) and Tris-buffered phenol, followed by centrifugation to remove protein-containing phenol fraction. Maize proteins were pelleted from phenol fraction by buffered methanol and resolubilized in dissolution buffer containing 8 M urea before reduction/alkylation and trypsin digestion.

In the more recent MRM assay (maize event MZIR260 study in 2024), the assay procedure was adapted from Birukou *et al*.^16^ Briefly, maize grain samples were extracted in phosphate-buffered saline containing 0.1% RapiGest^TM^, a mass spectrometry compatible detergent. Extracted proteins were denatured by adding equal volume of 2,2,2-trifluoroethanol and shaking before performing reduction/alkylation and digestion with trypsin.

In both MRM assays, samples were desalted prior to mass spectrometry analysis using either in-line reverse phase trap column or by off-line solid-phase extraction utilizing cation exchange resin to minimize matrix effects or interferences and reduce ion suppression. The digested peptides were chromatographically separated using reverse-phase chromatography with a gradient of acidified ACN in water. Both MRM assays were validated with respect to specificity, linearity, accuracy, and reproducibility in terms of quantification and comparison of the total LTP levels from samples in a study.

#### Data Analysis

Fifteen non-GM, commercial reference varieties were analyzed for total LTP content. These varieties were from commercial sources and were produced from different locations and years to provide a realistic representation of LTP content variability related to genetic and environmental factors. A tolerance interval (TI) was calculated from quantitative data as a statistical measure of the upper and lower bounds of the reference variety LTP concentrations across all 15 reference maize hybrid varieties. Briefly, the mean total LTP concentration for each reference variety was used to calculate a TI representing 90% of the population with 95% confidence (JMP statistics software, version 9.0.2; SAS Institute Inc., Cary, NC). The calculated TI of the 15 reference varieties is represented as a range with an upper and a lower expression level value in μg/mg, respectively.

For single-event varieties (Maize MZIR098 and Maize MZIR260), the LTP levels quantified with LC-MS/MS method for the test and control were compared using a Student’s *t*-test within each growing location. For stacked-event varieties, the quantified LTP levels for the test and control were subject to across-location ANOVA using the following mixed model:

$$Y_{\mathrm{ijk}}=U+T_{i}+L_{j}+B{\left(L\right)}_{\mathrm{jk}}+LT_{\mathrm{ij}}+e_{\mathrm{ijk}}$$

where *Y*_*ijk*_ is the observed response for entry *i* at location *j* in block *k*. The overall mean is represented by *U*, *T*_*i*_ is the entry effect, *L*_*j*_ is the location effect, *B(L)*_*jk*_ is the effect of block within location, *LT*_*ij*_ is the location-by-entry interaction effect, and *e*_*ijk*_ is the residual error. Entry was regarded as a fixed effect while location, block within location, and location-by-entry interaction were regarded as random effects. The analysis was conducted using the SAS® software v. 9.4 (SAS 2020).

## Results

### LTP Level Comparison with Western Blot Analyses

**Figure 1.**
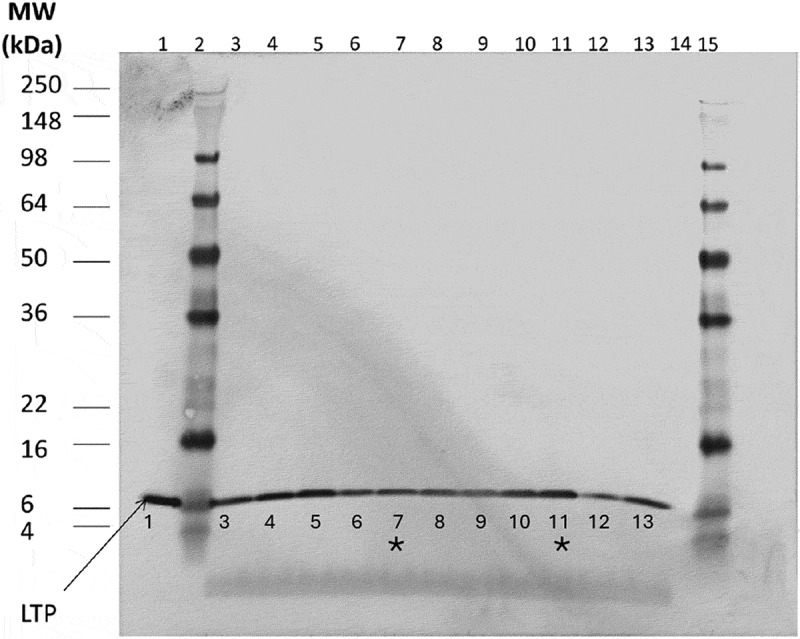
Western blot analysis of kernel extraction from Maize 5307 and Control and References.

When no absolute quantitation method was available, Western blot was initially employed to compare LTP levels in GM maize, their near-isogenic, non-GM control and conventional varieties from commercial source (“Commercial References,” or “References”). A transformation event 5307 generated a transgenic maize variety (“Maize 5307”). To measure the LTP level in the genetically modified Maize 5307, 5 μg total protein from its kernel extract was subject to Western blot analysis and compared with those from the kernel extracts of its near-isogenic, non-GM control variety as well as nine commercially available, non-GM reference varieties. Although many intrinsic variables may present to use Western blot to quantify proteins, the semi-quantitative comparison showed the band densities were similar across each sample, and the LTP band density from Maize 5307 (Lane #7, Figure 1) was not found to be elevated to a level significantly higher than its near-isogenic control (Lane #11, Figure 1) or other commercial, non-GM reference varieties, indicating the genetic modification did not have a significant impact on or alter the LTP level in the resulting Maize 5307 (Figure 1).

Purified LTP protein standard (200 ng) and a total of 5 μg protein from maize kernel extracts were loaded and subject to SDS-PAGE. The transferred proteins onto PVDF membrane were visualized with an alkaline phosphatase substrate solution after antibody probing. Lane #1, 200 ng standard; Lane #2 and 15, molecular weight marker; Lane #7, Maize 5307 and Lane #11, non-transgenic control for Maize 5307 were also labeled with the asterisk marks; Lane #3–6, 8-9, and 12–13, commercially available, conventional varieties; Lane #14, blank. The result was a representative of duplicated Western blot analysis.

**Figure 2.**
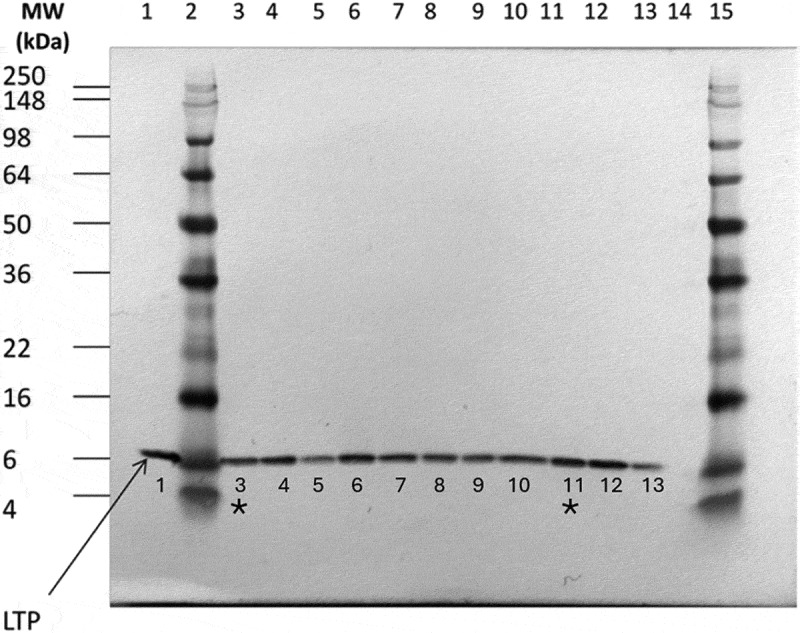
Western blot analysis of kernel extraction from maize 3272 × Bt11 × MIR604 × GA21, its non-GM Control and References. Purified LTP protein standard (200 ng) and a total of 5 μg protein from maize kernel extracts were loaded and subject to SDS-PAGE. The transferred proteins onto PVDF membrane were visualized with an alkaline phosphatase substrate solution after antibody probing. Lane #1, 200 ng standard; Lane #2 and 15, molecular weight marker; Lane #3, non-transgenic control for maize 3272 × Bt11 × MIR604 × GA21 and Lane #11, maize 3272 × Bt11 × MIR604 × GA21 were also labeled with the asterisk marks; Lane #4–10 and 12–13, commercially available, conventional varieties; Lane #14, blank. The result was a representative of duplicated Western blot analysis.

Similarly, such analysis was conducted on a stacked-event GM maize variety. Genetically modified single-event maize varieties, Maize 3272, Maize Bt11, Maize MIR604, and Maize GA21 were bred into a stacked-event GM maize variety, Maize 3272 × Bt11 × MIR604 × GA21 via conventional breeding methods (i.e., cross-pollination) in the same way as in breeding non-GM maize varieties. Each GM single-event maize variety was generated by modification of the maize genome via transformation to insert the designed foreign DNA construct so that the respective desired traits can be conveyed. None of the single-event maize GM varieties was designed or proved to have impact on endogenous LTP levels (data not shown). After they were stacked in the resulting Maize 3272 × Bt11 × MIR604 × GA21, the LTP level in the stack variety was compared with that in its near-isogenic control as well as those in non-GM commercial reference varieties (Figure 2). The semi-quantitative comparison in Figure 2 showed the LTP levels were similar across samples, and the LTP level in the stacked-event variety was not found to be elevated to a level significantly higher than its near-isogenic control or other commercial, non-GM reference varieties. The results indicate that breeding together GM single-event varieties did not impact on or alter the LTP level in the stack Maize 3272 × Bt11 × MIR604 × GA21.

### LTP Comparison with Mass Spectrometry: Single Transformation Events

**Figure 3.**
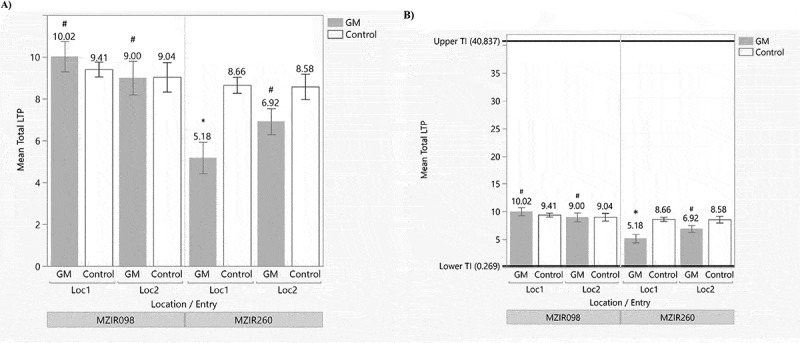
Comparison of LTP levels in genetically modified maize varieties, their non-GM Control and References. Panel a shows the side-by-side comparison of LTP levels from the GM maize varieties (“GM”) and their non-GM control variety (“Control”) at two growing locations for maize event MZIR098 and MZIR260, respectively. Panel B shows their comparison relative to the TI range with the upper and lower values determined from 15 commercial reference varieties. Data represented the mean values ± standard errors (SEM). All values are expressed in the unit of μg/mg. α = 0.05 was used in statistical comparison for determining any significant difference. #, representing no significant difference between GM and Control (*p* > .05); * representing significant difference between GM and Control (*p* < .05).

With the advancement of mass spectrometry technology, maize LTP contents were able to be quantified by LC-MS/MS method. The total LTP levels were quantified in MZIR098 and MZIR260 GM maize varieties, each of which contains a single transformation event. Their LTP levels were compared with those from their non-GM controls, respectively. As shown in Table 2, each GM variety was tested using kernel samples grown from two locations, and their LTP level (“GM”) was compared with their non-GM control variety (“Control”), respectively. The results showed MZIR260 GM maize had a significantly lower LTP level (*p* < .05) in comparison with its Control at one location but not at another location, while MZIR098 GM maize showed no statistically significant difference in the LTP levels from its respective control at either growing location (*p* > .05) (Table 2 and Figure 3). In addition, a total of 15 commercially available and non-GM reference varieties (“References”) were also quantified to establish an endogenous LTP reference range. The kernels from these reference varieties were produced from multiple locations and multiple years (Table 3). A TI was estimated with an upper and a lower concentration of 40.837 μg/mg and 0.269 μg/mg, respectively, representative of 90% of the population with 95% confidence. LTP contents from either GM varieties or their Control grown at two locations were all encompassed within the TI range (Figure 3).

**Table 2.** Comparison of LTP levels in genetically modified maize varieties, their non-GM control and references.

Variety	Location	Entry	Mean	SEM	Range	*p* Value
MZIR260	Germansville, PA	Control	8.58	0.609	6.87–9.69	
		GM	6.92	0.623	5.70–8.66	*p* > 0.05
	Sheridan, IN	Control	8.66	0.383	7.65–9.34	
		GM	5.18	0.752	2.98–6.37	*p* < 0.05
MZIR098	Bagley, IA	Control	9.41	0.353	9.04–9.89	
		GM	10.02	0.721	9.24–10.89	*p* > 0.05
	Larned, KS	Control	9.04	0.703	8.34–10.01	
		GM	9.00	0.802	8.21–10.12	*p* > 0.05
References			20.55	2.101	8.21–38.31	

Two GM maize varieties (“GM”) and their respective non-GM control (“Control”) were grown at two locations. LTP content in the kernels was quantified by LC-MS/MS method and compared with student t-test (α = 0.05) for statistical difference at each location. A total of 15 commercial reference varieties (“References”) were also measured for their LTP content with descriptive statistical results listed. All LTP concentrations were in the unit of μg/mg.

**Table 3.** Quantitation of LTP levels from commercial reference varieties.

Variety	Material ID	Location of production	Year of production	Mean of total LTP (μg/mg)	SEM	Range (min–max)
Reference 1	09BIC000001	Waterloo, NE, USA	2008	32.55	1.035	29.48–35.58
Reference 2	09BIC000002	Saint-Sauveur, France	2008	18.67	0.727	16.82–20.42
Reference 3	09BIC000003	Saint-Sauveur, France	2008	33.70	1.324	30.85––38.31
Reference 4	09BIC000004	Clinton, IL, USA	2008	33.43	1.248	30.60–36.49
Reference 5	09BIC000005	Waterloo, NE, USA	2008	21.00	1.587	17.50––24.81
Reference 6	09BIC000006	Phillips, NE, USA	2008	28.79	1.391	26.02––33.52
Reference 7	09BIC000007	Ames, IA, USA	2008	22.49	1.502	19.11––27.28
Reference 8	09BIC000008	Saint-Sauveur, France	2008	11.56	0.475	10.82––13.25
Reference 9	09BIC000012	Ames, IA, USA	2008	17.63	1.387	13.31––22.02
Reference 10	09BIC000013	Phillips, NE, USA	2008	18.83	1.033	15.96––21.43
Reference 11	09BIC000014	Slater, IA, USA	2008	18.17	0.850	16.14––20.07
Reference 12	09BIC000017	Pekin, IL, USA	2008	16.17	1.484	12.66––20.42
Reference 13	10ML009181	Graneros, Chile	2010	14.90	0.751	12.83––17.50
Reference 14	10ML009175	Graneros, Chile	2010	11.90	0.837	9.11––14.20
Reference 15	10ML009161	Graneros, Chile	2010	8.50	0.139	8.21––9.00

Kernels from a total of 15 non-GM conventional varieties that were commercially available were subject to LTP quantitation via LS-MS/MS method. The locations and years of production of maize kernels, and descriptive statistical results were summarized.

### LTP Comparison with Mass Spectrometry: GM Breeding Stacked Events

When stacked event varieties were examined, total LTP levels in kernels from 10 stacked maize varieties (Table 4, A–J “GM” in) were quantified and compared with their respective non-GM control (Table 4, A–J “Control”). These varieties were grown in multiple years at multiple locations together with non-GM commercial reference varieties (“References”) within the same studies, unless specified.

**Table 4.** Comparison of LTP levels in conventional breeding stacked GM maize varieties, their non-GM control and references.

Code	Stack event variety	Total LTP (μg/mg)					
		Entry	Mean	SEM	Range	p Value	
A	Bt11 × MZIR098 × DP4114 × NK603	Control	7.75	0.533	5.99–9.93	.410	
		GM	6.39	0.257	5.10–7.19		
		References	7.90	0.330	5.09–11.70		
B	Bt11 × MIR162 × MZIR098 × DP4114 × NK603	Control	7.25	0.704	4.33–10.98	.212	
		GM	7.58	0.680	4.99–10.24		
		References	7.01	0.323	4.12–10.37		
C	3272 × Bt11 × MIR162 × MZIR098 × DP4114 × NK603	Control	8.35	0.505	5.36–10.28	.250	
		GM	6.89	0.272	5.86–8.29		
		References	8.12	0.208	6.20–10.29		
D	3272 × Bt11 × MIR162 × MIR604 × TC1507 × 5307 × GA21	Control	10.92	0.140	10.20–11.53	.222	
		GM	10.24	0.376	8.69–11.43		
		References	9.35	0.265	7.16–12.30		
E	Bt11 × MIR162 × TC1507 × NK603	Control	7.25	0.704	4.33–10.98	.703	
		GM	7.45	0.710	4.85–10.44		
		References	7.01	0.323	4.12–10.37		
F	Bt11 × MIR162 × NK603	Control	7.03	0.353	5.15–8.45	.025	
		GM	8.36	0.256	7.55–9.53		
		References	7.90	0.330	5.09–11.70		
G	Bt11 × TC1507 × NK603	Control	7.75	0.533	5.99–9.93	.695	
		GM	7.34	0.218	6.73–8.44		
		References	7.90	0.330	5.09–11.70		
H	Bt11 ×MIR162 ×MIR604 ×MON 89,034 × 5307 ×GA21*	Control	7.46	0.320	6.35–9.02	.178	
		GM	9.53	0.250	8.32–10.44		
I	Bt11 ×MIR162 ×MON 89,034 ×GA21*	Control	7.14	0.362	4.87–8.22	.067	
		GM	11.39	0.621	9.04–13.21		
J	Bt11 × MIR162 × MON 89,034 × NK603	Control	8.88	0.740	6.39–11.84	.130	
		GM	9.51	0.589	7.33–11.83		
		References	8.12	0.208	6.20–10.29		

Summary of LTP level quantitation and comparison in 10 stack-event GM maize varieties (“GM”) versus their respective controls (“Control”) over a 10-year period. For each GM-Control pair, kernels were harvested from maize varieties that were grown at multiple locations together with commercial reference varieties in the same field trials (*, indicating reference varieties were not included in the same field trials). The LTP levels in the harvested kernels were subject to LC-MS/MS quantitation and subsequent statistical analysis. A mixed statistical model was used to perform an across-location ANOVA for comparison (α = 0.05, see “Materials and Methods” for details).

For the statistical comparison, an across-location ANOVA was performed with a mixed model (see “Materials and Methods”) based on the potential sources of variation in the field study design (entry [variety], location, block within location, and location-by-entry interaction). Entry was regarded as a fixed effect while location, block within location, and location-by-entry interaction were regarded as random effects. The model assures that the statistical comparison between entries (GM and Control) is not biased by variation expected from other effects in the model. The results indicated that no statistically significant differences were observed between 10 stacked-event varieties and their respective controls. Although a significant difference was observed between Bt11 × MIR162 × NK603 (F in Table 4) and its control (*p* < .05), the LTP levels for both Bt11 × MIR162 × NK603 (mean = 8.36 μg/mg, range of 7.55–9.53 μg/mg) and its control (mean = 7.03 μg/mg, range of 5.15–8.45 μg/mg) were within the range (5.09–11.70 μg/mg) observed from commercial reference varieties that were included in the same field trials.

**Figure 4.**
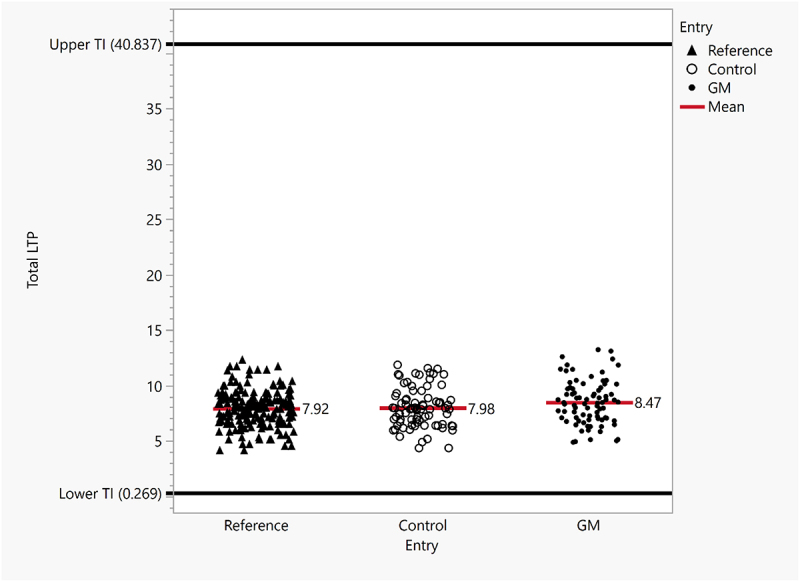
Comparison of LTP level distribution. LTP content from all tested maize stacked-event GM varieties, their respective controls as well as commercial references were displayed in the scatter plot. The TI was determined from the commercial reference varieties with the top and the bottom black lines representing the upper and the lower values, respectively. Each data point represents the mean value of samples from each location for the respective varieties. Each red bar represents the overall mean values for the respective entries (GM, Control, and Reference).

For an overall comparison, the distribution of the total LTP levels from each entry (Test, Control, and Reference) is displayed in a scatter plot (Figure 4). Each dot represents the mean value of an entry at a location, and the overall mean values were represented with the lines in the middle of the scattered dots with 8.47 μg/mg (*N* = 80), 7.98 μg/mg (*N* = 80), and 7.92 μg/mg (*N* = 192) for the Test, Control, and Reference, respectively. The results showed very similar distribution of the total LTP levels from each entry that were tested, and they were all well encompassed within the TI range. This was also observed from single-event GM varieties and their respective controls (Figure 3).

## Discussions

The LTPs are a family of small (7–9 kDa), basic, phylogenetically conserved proteins with widespread distribution across plant kingdom.^17^ These proteins are present extracellularly, often localized to cell walls.^17^ The LTP family proteins share a conserved and compact three-dimensional structure that displays stability in heat treatment and protease digestion.^18^ Although not completely understood, research work suggested LTPs play a role in pathogen defense in plants,^19,20^ and others suggested roles for plants to respond to abiotic stress.^17^

Several LTP family members have been identified as plant allergens. With Pru p 3 protein as a well-studied predominant LTP found in peach, LTP as a food allergen was well established.^18,21^ However, food allergy elicited by LTP sensitization has been reported with limited geographic distribution.^18,22^ Also, due to the conserved LTP structure, it is also not surprising that immunological cross-reactivity to botanically unrelated plant may occur.^23^ LTP in maize was identified as the major endogenous maize allergen from patients’ serological cross-reactivity to the protein,^9^ although few clinical cases were reported for maize induced anaphylaxis. With its long history of safe use in food and feed, maize is not considered a food allergen globally.

When maize was genetically modified, genes encoding LTPs or impacting LTP metabolism have never been a target for the genetic modifications (for historically approved GM maize, see relevant databases).^24,25^ However, considering any unintended impact on maize endogenous LTP from non-LTP-related genetic modifications, comparison of endogenous LTP levels between GM maize and its non-GM control was requested by a certain regulatory agency. In this study, we examined any possible impact of genetic modification on endogenous LTP levels in various GM maize varieties and compared them with their respective non-GM controls. In addition, various non-GM reference varieties from commercial sources that have been cultivated and consumed without any safety concerns were also included in the study to evaluate the natural range of the endogenous LTP levels in maize. The included GM maize ranged from varieties of a single-event transformation to varieties with multiple single events stacked in various combinations. The transgenes that have been inserted in maize genome have also varied including but not limited to genes encoding Bt (*Bacillus thuringiensis*) proteins for insect resistance traits, or enzymes for herbicide tolerance or starch hydrolysis. No gene thus far has been identified to have an impact on LTP metabolism in maize.

The study results indicate that transformation did not impact on endogenous LTP levels from either a semi-quantitative comparison via Western blot method or absolute quantitative comparison via LC-MS/MS method (Figures 1, 3 and Table 2). When single transformation events were combined via conventional breeding, the resulting stacked-event varieties were not expected to have an impact on endogenous LTP levels since none of the individual single event varieties had been indicated with such an impact from previous safety assessment outcomes^24,25^ (Figure 2, Table 4). This was furthermore demonstrated with more individual single events added into the combination (e.g., variety A to B to C, Table 4). Breeding individual maize transformation events into a combination is the same process as in conventional breeding of maize inherent traits from parent varieties (i.e., via cross-pollination). As a matter of fact, many countries do not require further safety assessment on a stacked GM variety provided the stacked variety was generated *via* conventional breeding, when the individual events for combination have already been assessed without any resulting safety concerns.^26,27^ Different trait combinations also showed no impact, for example, when an insect resistance trait was swapped with an herbicide tolerance trait (MIR162 in variety F and TC1507 in variety G, Table 4). The multiple GM and their respective non-GM control varieties used for quantitation and comparison of endogenous LTP levels were grown in multiple years and at multiple locations that are representative of typical maize cultivation areas. Thus, the data sets reflect real cultivation scenarios for maize cultivation. Taken together, the results from this study didn’t indicate any meaningful differences in the LTP levels between GM maize and their non-GM control varieties, indicating that genetic modification is not expected to impact endogenous LTP levels in GM maize.

The endogenous maize LTP level, like any endogenous maize proteins may vary greatly due to their genetic variability and various environmental factors such as nutrient and water availability, temperature, exposure to plant pathogens, harvest timing, and storage conditions.^28–30^ Therefore, a wide range of natural variability is expected to be found in non-GM maize. Using commercially available non-GM maize varieties as references, their endogenous LTP levels were measured and quantified, revealing a wide range of variabilities (Tables 2–4). A TI of endogenous LTP level was established from a total of 15 commercial reference varieties, suggesting ~150-fold possible normal distribution range (upper and lower levels of 40.837 and 0.269 μg/mg, respectively). Ranges of ~3.6-fold and 5.1-fold of endogenous LTP levels were measured and reported by other investigators with different quantitation methods.^15,31^ For the 10 stacked event varieties in the study, the LTP level from GM, non-GM Control and Reference had approximate mean values, and their distributions were well encompassed within the TI (Figure 4).

A long history of safe consumption of maize products has provided the evidence that human populations have been exposed to a wide natural variation range of LTP levels without allergy concerns.^11^ For LTP-sensitive populations, there is no known clinical cure for their sensitization to maize, and in such cases avoidance of any maize products is the best preventive measure to mitigate the allergy risk, regardless of whether it is GM or non-GM maize.^32,33^ When unintended effects from maize genetic modification are concerned, especially any potential risk that the endogenous LTP levels in GM maize may be increased to a level with elevated allergenicity risk, the targeted gene(s) for genetic modification have been and will continue to be carefully considered and thoroughly assessed. However, in practice, no GM crop developers had ever chosen LTP for genetic modification to avoid any potential concerns or risks (for reference, see https://www.isaaa.org/gmapprovaldatabase/default.asp). When genetic modification is not designed to specifically impact on endogenous LTP expression, it lacks a plausible mode of action that endogenous LTP levels will be significantly impacted on. For concerns about unintended effects of non-LTP related genetic modification, years of studies on GM maize LTP quantitation and comparison have not indicated any meaningful difference in endogenous LTP levels between the GM maize and its non-GM control, and the accumulated data also indicate any statistically observed difference, when observed, is very likely to be within the wide variations of LTP levels observed in non-GM maize. Therefore, quantifying and comparing LTP levels between GM and its non-GM control will not contribute any additional value to the allergenicity risk assessment of GM maize.
